# Current Application of Capillary Electrophoresis in Nanomaterial Characterisation and Its Potential to Characterise the Protein and Small Molecule Corona

**DOI:** 10.3390/nano8020099

**Published:** 2018-02-10

**Authors:** Andrew J. Chetwynd, Emily J. Guggenheim, Sophie M. Briffa, James A. Thorn, Iseult Lynch, Eugenia Valsami-Jones

**Affiliations:** 1 AB Sciex UK Ltd., Phoenix House, Lakeside Drive, Warrington, Cheshire WA1 1RX, UK; jim.thorn@sciex.com; 2 School of Geography Earth and Environmental Sciences, University of Birmingham, Edgbaston, Birmingham B15 2TT, UK; e.j.guggenheim@bham.ac.uk (E.J.G.); s.m.briffa@bham.ac.uk (S.M.B.); e.valsamijones@bham.ac.uk (E.V.-J.)

**Keywords:** capillary electrophoresis, nanomaterial, corona, mass spectrometry, biomolecules, characterisation, bio-nano interface, protein

## Abstract

Due to the increasing use and production of nanomaterials (NMs), the ability to characterise their physical/chemical properties quickly and reliably has never been so important. Proper characterisation allows a thorough understanding of the material and its stability, and is critical to establishing dose-response curves to ascertain risks to human and environmental health. Traditionally, methods such as Transmission Electron Microscopy (TEM), Field Flow Fractionation (FFF) and Dynamic Light Scattering (DLS) have been favoured for size characterisation, due to their wide-availability and well-established protocols. Capillary Electrophoresis (CE) offers a faster and more cost-effective solution for complex dispersions including polydisperse or non-spherical NMs. CE has been used to rapidly separate NMs of varying sizes, shapes, surface modifications and compositions. This review will discuss the literature surrounding the CE separation techniques, detection and NM characteristics used for the analysis of a wide range of NMs. The potential of combining CE with mass spectrometry (CE-MS) will also be explored to further expand the characterisation of NMs, including the layer of biomolecules adsorbed to the surface of NMs in biological or environmental compartments, termed the acquired biomolecule corona. CE offers the opportunity to uncover new/poorly characterised low abundance and polar protein classes due to the high ionisation efficiency of CE-MS. Furthermore, the possibility of using CE-MS to characterise the poorly researched small molecule interactions within the NM corona is discussed.

## 1. Introduction

Nanomaterials (NMs) are commonly defined as materials that have at least one dimension between 1 and 100 nm in size [1,2], and include nanoparticles (NPs) where all three dimensions measure <100 nm, as defined by European legislation including the cosmetics directive (2015/2283 [3]). In recent years, there has been a dramatic increase in NM publications and applications within a variety of different fields. This is partly due to their desirable chemical, electronic, optical, magnetic and mechanical properties, enhanced by, or due to, their nanoscale properties, which are distinctive from their bulk counterparts [1]. These properties are intrinsically linked to the composition, size and shape of the NMs. The same attributes that make them useful for applications can also be their source of potential risk due to enhanced reactivity and toxicity. Therefore, quick, accurate and cost-effective methods of characterisation are essential to thoroughly understand NMs, with sufficient throughput to allow time-resolved characterisation and sufficient accuracy to allow small changes in size distribution, including as a result of biomolecule interaction, to be determined.

To date, the most widely used techniques to characterise dispersed NMs are Dynamic Light Scattering (DLS), Transmission Electron Microscopy (TEM) and Field Flow Fractionation (FFF) [4] (Table 1).

DLS measures the size distribution of the NM suspension, or more specifically the hydrodynamic radius. The calculated result is indicative of the apparent size of the dynamic hydrated/solvated particle of a corresponding sphere [4]. However, due to this assumption, DLS cannot be deemed accurate for the sizing of non-spherical NMs. Additionally, a small number of agglomerated NMs or larger particles can mask the presence of smaller particles, as scattering scales with 1/D^6^. TEM is a microscopy method that can allow detection and resolution of individual NMs. However, it can be expensive and time consuming and the sample preparation may introduce artefacts such as agglomeration. Although only a small quantity of sample is needed for analysis, only a restricted amount of the prepared sample is actually imaged, reducing the representative analysis. Results are also subjective and very user dependant, with significant challenges for extracting useful information in agglomerated or aggregated samples. TEM analysis is also often done manually, which again is subject to operator bias and inter-user variability. FFF is a form of chromatography without a stationary phase. In FFF, NMs are separated based upon size, the mechanism of which has been extensively described [6]. Whilst FFF is very efficient and a relatively simple technique, sample recovery can be as low as 50% due to adsorption to the FFF membrane, thus reducing overall sensitivity of the analysis [6,7,11].

An alternative analytical technique is capillary electrophoresis (CE). CE offers great potential to characterise pristine NMs, biologically or environmentally aged NMs and the corona compositions acquired by NMs from biological or environmental matrices. CE offers a broad range of separation techniques such as capillary zone electrophoresis (CZE), micellar electrokinetic chromatography (MEKC), capillary gel electrophoresis (CGE), isotachophoresis (ITP) and capillary isoelectric focussing (cIEF). All of these CE techniques have their own unique separation characteristics and offer possibilities for the separation of proteins, small molecules and inorganic components such as NMs [12,13]. Another versatility of the CE system is that it is possible to use a range of detectors such as UV, Laser Induced Fluorescence (LIF), and various forms of mass spectrometry (MS). This means that a wide range of analyses can be performed using a single system and on a single NM sample [14]. The use of CE offers several benefits over the currently used techniques since CE can determine NM size in less than 20 min, is cost-effective, requires small reagent volumes, generates minimal waste and can separate complex mixtures of analytes [15].

A wide range of biomolecules are capable of binding to the surface of the NMs when exposed to complex environments, for example biological matrices such as blood. These adsorbed molecules at the NM surface are referred to as the NM (biomolecule) corona. This corona defines the way in which a NM is seen by cells within the body and can regulate the uptake and clearance of a NM, in addition to their transport around the body [16,17]. To date, most studies investigating the NM corona have focussed upon identifying specific NM-associated proteins which can bind to receptors and influence NM transport both around the body and within the cell [16,18]. This has been achieved using a wide range of techniques such as DLS, isothermal titration calorimetry (ITC), UV-vis and liquid chromatography mass spectrometry (LC-MS) [19,20,21]. The impact of corona formation on cellular uptake has been studied using traditional cell culture approaches, such as flow cytometric quantification of uptake, or confocal microscopy tracking of uptake of both NMs and their associated biomolecule coronas [22,23,24,25].

Another class of molecule thought to contribute to the NM corona are small molecules with a mass below 1000 Da, which thus far have received little attention. These molecules are collectively referred to as the metabolome, and can be produced endogenously or acquired from the environment (exogenous). An improved understanding of the dynamics of both the large and small molecules present within the NM corona may increase the understanding of NM transport, specifically into cells. This potentially opens the door for “designer” coronas to transport therapeutics to specific target areas and particular subcellular locations [16]. CE-MS offers a new approach to perform these analyses. CE-MS requires minute sample injection volumes (below 50 nL) while still offering a highly sensitive analysis due to the increased sensitivity of nano electrospray ionisation (nESI). Furthermore, this complementary orthogonal separation technique enables a different range of analytes to be investigated compared with conventional LC-MS.

The aim of this review is to discuss the fundamentals of CE for the NM community and to review the current literature surrounding the use of CE for NMs physico-chemical characterisation. This review spans from 1989 (first paper on CE separation of polystyrene nanoparticles) until 2017. Additionally, the potential to couple CE to high resolution mass spectrometers (HRMS) applied within the field of protein and small molecule corona characterisation will be examined and discussed, showcasing the potential for simultaneous characterisation of NM physico-chemical properties such as size and size distribution and corona composition in terms of small molecules and proteins.

## 2. Understanding CE as an Analytical Platform

### 2.1. CE Principals

**Figure 1:**
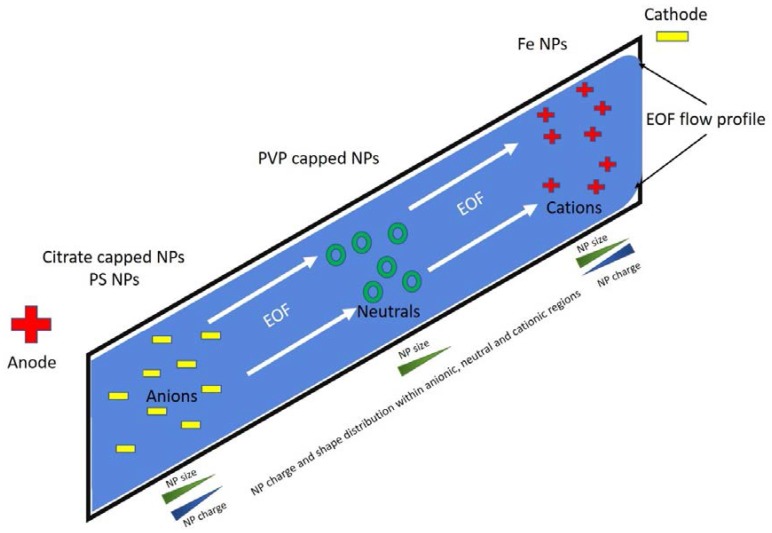
Capillary Zone Electrophoresis (CZE) analysis of nanoparticles (NPs) run in normal polarity mode. Cations are electrostatically attracted to the anode where the smallest and most charged arrive first; neutral analytes rely on the electroosmotic flow (EOF) to “push” them toward the anode and usually arrive from small to large. The negatively charged analytes are electrostatically attracted to the cathode but in cases where a strong EOF is present they can be “carried” toward the anode with the smallest size and lowest charge arriving initially followed later by the larger sized and highly charged NPs.

A CE system comprises a high voltage power supply, a capillary with an online detection window and an autosampler. The capillary is filled with an appropriate buffer; a sample is introduced at the end of the capillary, usually opposite the detector; and a high voltage is applied to the ends of the capillary. CE offers a broad range of dynamic separation modes. Each of these has its own niche, which, unlike liquid chromatography (LC), can all be achieved using a bare fused silica (BFS) capillary either in its raw form or with the addition of a dynamic/non-dynamic coating to change its surface chemistry [26]. CE separation takes place in this capillary, with both ends submerged in a buffer reservoir and an electrical voltage applied. Separation is a result of the different mass to charge ratios of each analyte in the background electrolyte (BGE) solution under high voltage [27]. Charged analytes migrate toward their respective electrodes; (+) cations to the (−) cathode and (−) anions to (+) anode. When a BFS capillary is used, the negative surface charge of the silanol groups attracts cations to form a diffuse double layer, creating a potential difference close to the capillary surface. Under high voltage these cations are propelled towards the cathode, dragging the bulk flow of solvent with them to form an electroosmotic flow (EOF) towards the cathode. At pH > 7, the EOF is fast enough to sweep cations, neutral analytes and anions towards the detector. A depiction of CZE separation as applied to NMs is shown in Figure 1. This means that all analytes can be detected in a single injection. However, there are some instances where an EOF is undesirable and as such a dynamic/non-dynamic coating can be applied to the BFS capillary to eliminate the surface charge of the capillary and thus greatly diminish, or remove entirely, the effect of EOF [12,26].

### 2.2. CE Separation Techniques as Applied to NM Characterisation

**Figure 2:**
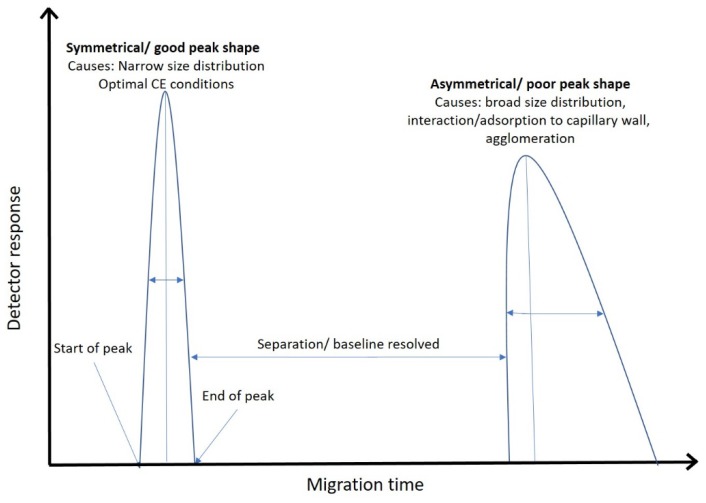
A hypothetical electropherogram to illustrate chromatographic separation and peak shape.

The two most commonly used CE techniques for the separation of NMs are MEKC and CZE, which are briefly described here. CZE is the most basic and commonly used form of CE separation. Here, the sample is injected into a buffer filled capillary and a high voltage applied. Analytes are separated based upon charge and size, with each of these properties determining the velocity at which they move through the capillary to the detection window. Typically, small highly positively charged NMs would elute prior to neutral NMs which are carried through the capillary via the EOF, followed by the smaller less negatively charged NMs where the EOF overcomes the anodic attraction (Figure 2). MEKC separation is typically based upon differential partition coefficients of analytes between micelles (acting as a pseudo stationary phase) and running buffer [27,28]. In these analyses, a BFS capillary is used with a high pH BGE to create a high EOF for fast run times. The formation of micelles for the analyte to partition into requires a surfactant to be present in the running buffer, the most common of which is sodium dodecyl sulphate (SDS). SDS needs to be present at a concentration greater than its critical micellar concentration (SDS ≈ 8.2 mM in water) for micelles to form. The structure of these micelles means that the hydrophobic component of SDS is present on the inside, and the negatively charged headgroups are at the surface of the micelle [28]. Therefore, the micelles migrate by electrophoresis away from the detector and against the EOF. NMs that partition into the micelles will thus be delayed reaching the detector by the counter-migration of the micelles. Thus, the greater the interaction the NMs have with the micelle the longer their migration time. NM separations however are more likely to result from SDS molecules interacting with the NM surface. Larger NMs can interact with more SDS monomers therefore imparting a greater overall negative charge, thus affecting the NMs electrophoretic mobility, enabling them to resist the EOF more than a small particle with fewer SDS interactions. Consequently, larger NMs migrate to the detector at a slower rate than small NMs, generating size based separation. In these separations, the elution order is the opposite of CZE, as negatively charged NMs would remain in solution due to electrostatic repulsion of the negatively charged SDS, neutral NMs would then elute solely as a function of their hydrophobicity, whereas positively charged NMs will be the last to elute due to their interaction with the negatively charged SDS/micelles thus giving them a large overall negative charge meaning that the EOF must overcome the electrostatic anodic attraction of these NMs [28]. In some studies, an EOF/neutral marker such as methanol is incorporated into the sample buffer, which does not partition into the micelle and moves with the EOF, signalling the beginning of the separation window. In addition, a micelle marker such as Sudan III can be used which is completely incorporated into the micelle and elutes at the end of the separation window [28].

In both techniques, the concentration and pH of the buffers used are vital to achieve high quality separation and peak shape. A number of studies have shown that increased concentrations of SDS in MEKC studies improve resolution between peaks and increase migration times on the electropherogram by increasing mobility within the capillary [29,30,31]. While the buffer salt concentration can be increased, thus increasing the ionic strength of the buffer to reduce the analyte migration time, this is at the risk of peak broadening and reduced resolution due to joule heating, excess heat due to a high current, and reduced EOF [31,32,33]. The effect of pH on peak shape and distribution is well established: In MEKC, a more basic pH reduces run times and separation efficiency as a result of an increased EOF [30,32]. The use of organic solvent in the separation buffer can increase resolution as it reduces the EOF. However, these solvents can also lead to current dropouts during the analysis thus causing poor quality data to be acquired [34,35].

### 2.3. CE Detectors for NM Detection

#### 2.3.1. CE-UV

One of the advantages of using CE is that the technique can be coupled to a range of different detectors. UV is the most common detector used when characterizing NMs, as it can detect both UV absorbing and non-absorbing NMs. While UV also allows for the quantification of analyte, the sensitivity is limited to the mg/L range [36,37]. CE-UV has been utilized for spherical, rod and tube NM size determination, since CE mobility and NM size are directly proportional. Therefore, very good *R*^2^ values can be obtained making the determination of an unknown NM size relatively simple [31,38,39,40]. NM composition, to some extent, can be differentiated through mobility or specific UV absorption spectra. However, prior knowledge of the NMs is necessary, as CE-UV alone cannot identify the composition of unknown NMs. NM size can also be analysed using a range of other optical detectors, such as LIF and DLS, by constructing size based calibration curves and calculating the NM size [34,41,42]. These techniques generally have greater sensitivity than UV-vis [41,43,44].

Several studies have also used CE-UV to calculate many other NM properties (see Table 2), most of which require the electrophoretic mobility of the NMs to be calculated. In a typical electropherogram the apparent mobility is observed. This is the combination of the electrokinetic mobility, i.e., the mobility of the NM within an electric field, and the electroosmotic mobility, i.e., the mobility of the NM as an effect of the electroosmotic flow [45,46]. The calculations for electrokinetic mobility are described below, based on CE separation of NMs [35]:

(1) $$\mu_{e}=\frac{1}{∆E}\left(\frac{L}{t_{e}}-\frac{L}{t_{o}}\right)$$

Here, *µ_e_* represents the electrophoretic mobility, Δ*E* the potential gradient across the capillary, *t_e_* and *t_o_* the migration time of the NM and the neutral marker/EOF, respectively, and *L* the effective capillary length. Calculating the electrokinetic mobility enables a wide range of NM properties to be determined, such as the direct calculation of a NM’s zeta potential [47]. This calculation has been shown to be comparable to conventional methods of zeta potential determination for gold and silica NMs [47,48]. Using this calculated zeta potential, it is then possible to determine the surface charge density of the NM; the details for these calculations have been well described for gold and silica NMs [47,48,49].

The hydrodynamic size can be determined using Taylor Dispersion Analysis (TDA). TDA involves the injection of a sample into a laminar flow of matched buffer to produce a Taylorgram and the measurement of peak broadening over time, thus two detection windows are required [50]. This facilitates determination of the analytes’ (e.g., the NMs’) diffusion co-efficient, consequently allowing its hydrodynamic size to be calculated. This has been widely discussed and demonstrated in several studies [47,48,49,51]. Briefly, to determine the hydrodynamic size, several calculations are required based upon initial migration time measurements (Equations (2)–(4)). Initially, the temporal variance is determined using Equation (2):

(2) $$\sigma =\frac{\sum_{i=n}^{i=m}h_{i}{\left(t_{i}-t_{d}\right)}^{2}\left(t_{i+1}-t_{i}\right)}{\sum_{i=n}^{i=m}h_{i}\left(t_{i+1}-t_{i}\right)}$$

where *σ* is the temporal variance, *h_i_* and *t_i_* are the detector response and the migration time at a given point on the Taylorgram, *n* and *m* are the beginning and end of the peak and *t_d_* is the average migration time for the peak. The value determined for the temporal variance can then be input into Equation (3) to calculate the diffusion coefficient:

(3) $$D=\frac{R_{C}{}^{2}}{24\sigma^{2}}t_{d}$$

Here, *D* is the diffusion coefficient, *R_c_*^2^ the radius of the capillary and *t_d_* the migration time. Using this calculated diffusion coefficient, the Stokes–Einstein equation (Equation (4)) can be used to determine the hydrodynamic size of the NM.

(4) $$R_{h}=\frac{k_{B}T}{6\pi \eta D}$$

where *R_h_* is the hydrodynamic size, *k_B_* is the Boltzmann constant, *η* is the solvent viscosity and *T* is the absolute temperature.

#### 2.3.2. CE-MS for Elemental Analysis

A detector that is becoming more frequently attached to CE is inductively coupled plasma–mass spectrometry (ICP-MS). To date, CE-ICP-MS has been performed using a quadrupole detector within the MS allowing a small number of elements to be analysed at any one time. However, the development of ICP-Time of Flight-MS (ICP-TOF-MS) offers the potential for multi-element analysis of complex mixtures of NMs and may in the future be hyphenated to CE technology [52]. These MS detectors determine the elemental composition of NMs with detection limits of ng/L, making them ideal for NM detection in more complex media such as blood plasma [36], but also for confirmation of the elemental composition of the NMs which was one of the limitations identified above for optical detectors. The MS detectors can be used in a similar manner to CE-UV to create a calibration curve for NM size or, more commonly, the ICP-MS response can be used to determine the size of the NM [14,34,36]. Furthermore, ICP-MS can be modified to have a microsecond time resolution, allowing single NMs to be detected, e.g., against a background of ions of similar elemental composition [52,53]. This method is termed single particle ICP-MS (spICP-MS). Here individual MS spikes above the background represent a NM and the number of counts within the spike is correlated to the NM size [54]. This method can be used to calculate the average NM size, size distribution and particle number concentration (PNC) [52,53,54], as well as particle density, and morphology of complex bimetallic NMs such as core-shell or chemically doped mixed-phase NM (Table 2).

## 3. Summary of NMs Analysed by CE to Date

### 3.1. Gold NPs

To date, gold NPs (AuNP) have received the most interest from groups using CE, potentially due to their strong surface resonance properties which make them easily detectable by UV [27,55]. The physical and chemical properties of AuNPs also render them useful for applications such as the enhancement of Raman scattering in surface enhanced Raman spectroscopy (SERS) [56], imaging [57,58], cancer cell detection [58], drug delivery [58] and as anti-viral agents [59].

**Figure 3:**
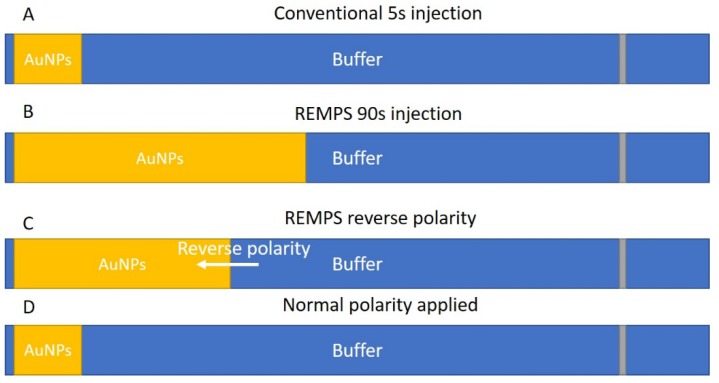
Schematic of reversed electrode polarity stacking mode (REPSM): (**A**) standard 5 s 50 mBar injection; (**B**) larger REPSM injection of 90 s fills a large proportion of the capillary; (**C**) application of a reverse polarity separation voltage concentrates the sample toward the inlet of the capillary; and (**D**) once the sample has been concentrated, a normal polarity separation is performed but with a much greater sample loading than in a conventional injection.

The most widely used CE-based method for size separation of spherical AuNPs is MEKC [29,30,31,38]. Normally, this separation modality is coupled to a UV detector. However, Lo et al. in 2008 used a CE linked to an LIF detector to improve detection limits. These methods have shown that AuNPs can be separated by size over two orders of magnitude, ranging from 3.5 nm to 59.9 nm. They also demonstrated that mobility is directly proportional to AuNP size, with *R*^2^ values of 0.985–0.999 and highly reproducible migration time relative standard deviations (RSDs) of <1% [31,38,60,61] (Table 3). In these studies, the separation buffer was typically composed of 70 mM of the surfactant SDS and 10 mM of the salt 3-cyclohexylamino-1-propanesulphonic acid (CAPS) adjusted to pH 10 (Table 3). This choice of buffer concentration is a compromise between the quality of peak resolution, peak symmetry and separation (Figure 2) afforded by a higher concentration of SDS versus the desire to reduce run times by lowering the SDS concentration [30,31,32,38]. Furthermore, the addition of a salt such as NaH_2_PO_4_ to the AuNP solution reduced the EOF and subsequently increased separation and theoretical plate count at 10 mM. However, further increases in NaH_2_PO_4_ concentration dramatically increased peak width and reduced theoretical plate count [32]. An additional step has also been used which concentrates the sample prior to separation; this allows more sample to be injected while reducing the length of capillary the sample fills prior to separation [61]. This method is termed reversed electrode polarity stacking mode (REPSM). In REPSM, immediately following a large injection, the capillary inlet is placed into the separation buffer and a reverse polarity potential is applied. This concentrates the sample towards the “inlet” prior to a normal polarity separation (Figure 3). It is important to consider the sample solution in REPSM: in order to achieve this concentration effect the sample must be solubilised in a low conductivity solution relative to the highly conductive MEKC BGE [62]. This technique has been shown to increase CE-UV sensitivity to AuNPs by 10 to 500 fold as more sample is loaded onto the capillary with no detriment to separation [30,32,61].

A single paper has utilised MEKC (70 mM SDS, 10 mM CAPS) CE separation prior to analysis with spICP-MS for AuNPs (Table 3) [52]. This technique enables individual AuNPs to be detected and subsequently calculates their average size, size distribution and PNC [53,54]. The coupling of CE to spICP-MS effectively doubles the size resolution as both hydrodynamic size and analyte mass can be ascertained [52]. Furthermore, the CE separation prior to spICP-MS separates the AuNPs from both dissolved Au ions and matrix, thus enhancing the size detection limit by minimising background signal [52]. This study achieved separation of 10, 30, 60 nm AuNPs via CE and allowed for migration time, size and PNC to be determined in a single run. In addition it was demonstrated that the size determined using CE-spICP-MS was in agreement with the current gold standard method, namely Electron Microscopy (either Transmission or Scanning) [52].

Several studies have also incorporated a CZE separation approach for AuNPs. In one study by Schnabel et al., a buffer of 6 mM sodium acetate/acetic acid at pH 5 was used to analyse 5 spherical AuNPs ranging from 5.2 nm to 14.6 nm (Table 3). As with the MEKC methods described above, there was a high correlation between mobility and NP size with an *R*^2^ of 0.9745. The authors found that a lower buffer concentration decreased NP mobility and thus reduced separation [39]. A comprehensive analysis of a range of spherical AuNPs sized 4 to 20 nm was performed using CE-TDA to determine hydrodynamic size, zeta potential and effective charge numbers in a single 25-minute run. These results were then compared to conventional measurement methods (DLS) and found to be in good agreement [48]. The coupling of TDA to CE is particularly beneficial as CE characteristically has a low zone dispersion (little dispersion into the surrounding buffer). This allows for a less complex calculation of size distributions compared with FFF, which has greater dispersion and also loses some sample to the FFF membrane [48]. A further study used CE with an evaporative light scattering detector (EVLSD) to characterise low levels of AuNP. Here, CZE was chosen over MEKC to reduce background noise associated with micelles. This study utilised a buffer of 20 mM tris(hydroxymethyl)aminomethane (Tris), ammonium acetate and 10 mM CAPS at pH 8.5 which separated 3.5, 6.5 and 10.5 nm AuNPs, although not baseline resolved, highlighting the high degree of separation achievable with CE [42].

A CZE separation has been used for direct comparison of UV, ICP-MS and conductivity detectors. The ICP-MS offered the lowest limit of detection (LOD), with <µg/L detection limits, thus enabling more environmentally relevant levels of AuNPs to be analysed. When comparing the UV and conductivity detectors, it was found that the dynamic concentration range of the conductivity detector was greater than that of the UV detector. However, with the conductivity measurements no separation voltage was applied. Instead the sample was pushed towards the detector using pressure. When used under standard CE conditions with a separation voltage, no response was detected using conductivity [14].

### 3.2. Silver NPs

Silver NPs (AgNPs) have received considerable interest due to their antibacterial, electrical, optical and oxidative properties compared to bulk silver [55,63]. For this reason they have been incorporated into coatings, fibres, bandages, dressings, plasters, plastics, soap, textiles and cosmetics [55]. MEKC has been utilised to separate AgNPs; as with AuNPs, Liu et al., were able to distinguish these NMs based upon both their size and shape. Using a 10 mM Tris based running buffer with SDS at 20 mM they could separate 17 nm and 49.7 nm spherical AgNPs within 15 min (Table 3). Although this buffer system also facilitated separation of nanospheres and nanorods, it was not baseline resolved. It was however possible to discriminate between the two shapes using the UV detector, based upon their different plasmon surface resonance [63].

In 2015, another form of CE separation, called isotachophoresis (ITP), was applied. Here, the NPs were injected between a leading and terminating electrolyte of different ionic strengths to focus the sample [40]. To reduce the EOF in the system, a BFS capillary was coated with fluorinated ethylene–propylene copolymer to create a neutral capillary (i.e., a capillary with no surface charge). Here, AgNP colloids stabilized in gelatin were separated based upon size using two CE methods set at different pHs, 7.1 and 4.5. The latter of the two methods provided much greater electrophoretic resolution. In addition the *R*^2^ value for the relationship between AgNP size and CE mobility was greater at the lower pH (0.9758 at pH 7.1 versus 0.9954 at pH 4.5) [40]. This method enabled complete baseline separation of 4, 10, 16 and 22 nm spherical AgNPs within 25 min, representing a significant improvement on the standard methods.

Recently CE has been coupled to spICP-MS for AgNPs analysis in two studies. The first study showed proof of principal by separating 20, 40 and 60 nm AgNPs and demonstrated that a REPSM pre-concentration step can be incorporated. This lowered the effective detection limits 14.3–27.7 fold, thus significantly improving the ability to detect <µg/L levels of NPs with an already very sensitive detector [53]. The second study demonstrated that the surface coating of NPs can cause significant differences in AgNP migration, a property that had been over looked in similar studies. Here, 40 nm AgNPs with 4 different coatings, i.e., branched polyethyleimine (BPEI), polyethylene glycol (PEG), polyvinylpyrrolidone (PVP) and citrate, were found to elute in order of the charge of their coating, starting with the positively charged BPEI and ending with the highly negative surface charge of citrate. However, with the 20 nm particles it was not possible to separate the citrate and PVP coated AgNPs, this is potentially due to there being very little difference between their size: charge ratio with a zeta potential difference of only 6.4 mV and average diameter difference of 2.17 nm. It may have been possible to further optimise the buffer system, however, the desired short run time may not then have been possible. Furthermore, in a complex environmental sample, 20 nm citrate capped and 40 and 60 nm PVP capped AgNPs co-eluted and were thus not identifiable using CE separation only, again potentially as a result of the chosen buffer system and the desired run time. Using the ICP-MS in standard mode it was also not possible to distinguish between these differently sized and capped particles and only when used in spICP-MS mode with the microsecond scan resolution was it possible to distinguish the PVP from the citrate coated NPs [54].

### 3.3. Carbon NMs

Carbon NMs come in several forms such as single- or multi-wall nanotubes (SWNT and MWNT, respectively), buckminsterfullerenes (or buckyballs—C_60_ and C_70_) or more recently graphene sheets, each of which pose their own unique challenges for characterisation in general, and for CE characterisation specifically. The physical structure of carbon nanotubes (CNTs), rather than their chemical properties, plays a larger role in deciding the optimal CE separation technique. Typically, C_60_ and C_70_ fullerenes are separated by MEKC [64,67,70] while nanotubes are separated by CZE as these are typically too “long” to fit within a micelle (Table 3) [67]. Typically, all carbon NM samples are dispersed in SDS prior to analysis to avoid agglomeration [67]. Carbon NMs pose an interesting problem as they are insoluble in water. The function of SDS is thus twofold with these NMs. Not only does the SDS prevent agglomeration and help disperse the NMs, but the formation of micelles also “solubilises” them [67]. In the case of buckyballs, MEKC is typically used as the separation method and can generate baseline separation of both C_60_ and C_70_ in less than 20 min. However, relatively high concentrations of SDS are often implemented (up to 150 mM) to increase both the sensitivity and resolution. This requires a lower separation voltage be used to reduce joule heating [64,70]. Interestingly, Chan et al. developed both a CZE and MEKC method for the analysis of fullerenes in human serum. In this study, a method to separate and quantify dendrofullerene and carboxyfullerene spiked human serum was developed, with detection limits of 0.5–6.0 µg/mL, paving the way to determine human exposure to these substances [64].

Both SWNT and MWNT are separated using CZE methods with ammonium acetate, trizma base or glycine as a running buffer in basic conditions [65,66,67,69]. When CNTs dispersed in SDS are run using a CZE method, the CNTs are negatively charged and migration to the anode is via the EOF. A range of CNTs have been separated based on size, 0.7–1.2 nm × 2–20 µm to 5–20 nm × 20–50 µm, or based on PVP coating chain length from 20 nm to 1 µm [66,69]. In these studies CE is shown to separate CNTs based upon not only length but overall shape, diameter and cross sectional area [66]. These methods using CE have also been demonstrated to have improved size selectivity when compared to size exclusion chromatography and FFF, and can be used to purify CNT solutions [67].

Graphene has received considerable interest in recent years due to its electrical properties. Therefore, it has started to receive attention for characterisation by CE (Table 3). Thus far, two studies have investigated the separation and detection of graphene oxide (GO) using CZE on BFS capillaries [68,71]. In 2010, a method was developed to analyse GO and chemically converted GO (CCG, also called reduced GO), whereas a second study looked solely at GO. In both cases, buffers of low ionic strength were required to minimise aggregation. Muller et al., used 250 µM tetrapropylhydroxide acid as opposed to the 2 mM borate buffer with a greater ionic strength used by Zhao et al. [68,71]. Unlike in other NM studies, the addition of SDS to the sample buffer did not appear to reduce aggregation [71]. As with other NMs, the GO sheets eluted from the capillary in order of size with the smallest first, and single flat sheets appear to elute before double or triple folded stacks, thus enabling the purity of single sheet GO to be determined [71].

### 3.4. Polystyrene NPs

Polystyrene (PS) NPs have been used within a variety of applications such as photonics, biosensors and biomedicine/nanotoxicology [76]. PS NPs are attractive for study as they are widely available, homogenous, can form stable colloids in biological fluids and are thought to be predominantly biologically inert [75,76]. PS NPs are well studied and there is a breadth of information available regarding their properties and effects and, a range of different surface-modified PS NPs are available as well as numerous fluorescently-labelled variants [77,78]. The separation of PS NPs was one of the first applications of CE within the field of NMs research. In 1989 a CZE method was developed for the separation of 39 nm, 72 nm, 132 nm, 308 nm, 488 nm and 683 nm PS NPs within 6 min using a borate buffer system (Table 3). In this study, the BFS capillary was rinsed with cetrimonium bromide (CTAB) surfactant to reduce the interaction between the NPs and the hydrophobic sites on the BFS capillary. This method proved to be highly reproducible for migration time with an RSD of 1.4% (*n* = 8) and showed again that mobility in CE is proportional to NP size for spherical NMs [33]. Since then, a basic (pH 9.2) phosphate buffer system has been used to separate NPs ranging from 50 to 600 nm in less than 15 min [72]. Another similar study achieved separation of PS NPs between 20 and 300 nm in size using a Tris buffer system at pH 9.2 [45]. More recently CE was used to perform TDA where 3 UV windows were implemented. Here, NP absolute size was compared to that calculated by DLS, standalone TDA and CE-TDA. The study showed that there was a high degree of agreement between the 3 techniques for the 2 PS NP sizes studied. Furthermore on the same CE system baseline separation was achieved for the 71.5 nm and 54 nm NPs, thus giving a secondary measurement of size if a calibration curve had been generated [51].

The combination of CE linked to a laser light scattering detector (CE-LLS) has rarely been documented; with only one published study thus far for NM characterisation. The use of LLS provides a means of detecting single unmodified NPs. In the study, a Tris, borate, and ethylenediaminetetraacetic acid (EDTA) buffer system was used to achieve baseline separation using CZE of 57 nm, 110 nm, 202 nm, 336 nm, 548 nm, 754 nm, and 992 nm PS NPs using an electrokinetic injection; this separation was achieved in less than 10 min. When used as a single particle detector, the size of the NP was found to be proportional to the intensity of the LLS response and was detected with an efficiency of 38–57%. This technique offers a significant improvement on detection limits for NP analysis when compared to UV where 2000 683 nm NPs or 3.6 million 39 nm NPs are required for detection compared to a single NP using LLS [79].

### 3.5. Silica NPs

Silica NPs (SiNPs) have been used as an approved food additive (E551), which is an anti-caking agent, for several years [34,47]. The two studies to date investigating pristine silica NPs with CE utilize CZE separation. In the first study, a CZE method was developed because evaporative light detection was used and MEKC micelles cause high levels of background noise [34]. In this study a near baseline separation was achieved for 20 nm, 50 nm and 100 nm silica NPs within 15 min with LODs and limits of quantification (LOQs) of between 1.08–1.11 ng/µL and 1.3-1.46 ng/µL, respectively. Furthermore, the method proved to be very reproducible between injections, with an RSD for peak area of between 4% and 5.7% for migration time. The low LODs and LOQs achieved enable the method to quantify SiNP levels in environmentally relevant samples such as foodstuffs, in addition to determining NP properties following synthesis [34]. The second study utilised CE-UV to perform TDA and achieved full baseline CZE separation of 7 nm, 12 nm and 22 nm SiNPs in a very reproducible manner with an RSD of 0.74% for migration time. Furthermore, by using this mobility data within the CE capillary, they mathematically calculated the zeta potential and surface charge density [47]. The values calculated compared favourably with values determined using traditional instrumentation. This highlights the potential CE has for determining a wide range of NP properties, thus reducing the number of experiments required to characterise these NPs [47].

### 3.6. Iron NPs

Iron (Fe) NPs have a wide range of applications from environmental remediation to their potential use as contrast agents in biomedicine [80,81,82]. This is due to their advantageous properties including, but not limited to, assumed biocompatibility and magnetism. When ferromagnetic Fe NPs have a core size of less than 30 nm they exhibit different magnetic properties, termed superparamagnetism. Superparamagnetic NPs no longer show permanent magnetisation; instead, magnetisation can be induced by externally applied fields [83]. More importantly, upon removal of the external magnetic field, no residual magnetisation remains. This is important for prevention of agglomeration within biological systems such as blood vessels [84]. These so-called superparamagnetic Fe NPs (SPIONs) offer immense potential within diagnostics and therapeutics. Their magnetic properties render them good negative contrast enhancers in Magnetic Resonance Imaging applications, allow magnetic guiding to target sites of interest, field modulation of drug release at target sites, in addition to enabling their use for external magnetic detection [81,85,86,87,88].

Due to the positive surface charge of Fe NPs, and those stabilised with HNO_3_, the negatively charged surface of BFS capillaries needs to be modified to prevent Fe NP adsorption to the capillary wall and resultant loss of sample. To mitigate this, an investigation into different possible capillary coatings was carried out by d’Orlye et al. In this study, three coatings were investigated for use in a CZE separation: a hydrophilic neutral coating, i.e., hydroxypropyl cellulose (HPC); a polycation coating, i.e., hexadimethrine bromide/polybrene (PB); and a double chained cationic surfactant didodecyldimethylammonium bromide (DDABr). These were tested with a HNO_3_ stabilised Fe NP size series of 6.8 nm, 8.9 nm and 10.6 nm, with each size injected separately. It was found that HPC supressed the EOF but did not prevent FeNPs adsorbing to the capillary wall, thus adversely affecting peak shape and reducing the amount of sample detectable. The PB coating generated an anodic EOF but the analysis suffered from very poor peak shape because of the Fe NPs interacting with the capillary wall. The DDABr coating returned symmetrical peaks suggesting minimal, if any, interactions with, or adsorption to, the capillary wall. In addition, this capillary coating provided a high anodic EOF thus reducing run times for the size series to < 4 min compared to the 5 min for HPC and 16 min for PB coated capillaries. The type of capillary coating also had a significant effect on the mobility of the Fe NPs in the capillary. When the HPC coated capillary was used Fe NPs migrated in size order from smallest to largest, whereas with the PB and DDABr capillary coatings the migration order was reversed. Regardless of the capillary coating used, the migration time reproducibility was very good with RSDs of <1.1%. This study also demonstrated that when Fe NPs are stabilised with either citrate or tetramethylammonium hydroxide (TMAOH), thus giving the Fe NPs an overall negative charge, an unmodified BFS capillary can be used and achieves separation of 7 nm, 8.9 nm and 10.3 nm citrate capped or 6.8 nm and 8.8 nm TMAOH capped Fe NPs when each NP group was injected separately (Table 3) [73].

A follow-up study by the same group used CE-UV to assess the functionalisation of Fe NPs. Here again the DDABr coated capillary was implemented to prevent positively charged functionalised Fe NP interactions with the capillary wall. Here maghemite particles were manufactured with a silica shell to allow functionalisation via 3-(aminopropyl)triethoxysilane (APTES) and 2-[methoxy(polyethyleneoxy)propyl]trimethoxysilane (PEOS) to form an amino-PEGylated functionalised Fe NP. In this study, eight Fe NP groups were manufactured, all of the same size. These differed in surface charge density by changing the molar ratio between APTES and PEOS. CE separation by CZE using a 106.6 mM Tris/100 mM hydrochloric acid (HCl) BGE enabled separation based upon APTES: PEOS ratio/surface charge density. When compared to the ninhydrine colorimetric assay which is the current gold standard test for surface charge density, there was a good linear relationship with an *R*^2^ value of 0.90. Furthermore, the CE method proved to be much more reproducible with an RSD of <1.5% compared to 54% for the ninhydrine assay (Table 3) [74].

### 3.7. Analysis of Mixtures of NMs

In environmental samples, it is highly unlikely that only one species of NM will be present. Therefore, methods capable of detecting and differentiating between NMs of different compositions, shapes and dimensions are required. One such method utilised CE-UV and a CZE separation to analyse a mixture of Au and PS NPs. In this study, both migration time and UV response were used to differentiate particles based upon both their size and composition. This indicates that CE-UV can provide a cost effective and high throughput analysis of NMs mixtures in terms of composition and size [45]. Another method which promises to distinguish between almost any species of NM is CE-ICP-MS where both CE and ICP-MS can be used to separate NMs by elemental composition. The separation of Ag and AuNPs has been the most investigated to date. In one study, a MEKC separation using 60 mM SDS and 10 mM CAPS buffers to provide baseline separation of 5 nm, 20 nm and 50 nm AuNPs was achieved with an *R*^2^ of >0.999 for the correlation between NP size and mobility. The capacity to carry out simultaneous Ag and AuNP analysis was also investigated using 10 nm and 30 nm NPs of each composition. While separation of the 30 nm particles was possible using the CE method alone the ICP-MS was able to distinguish between the two compositions and sizes [36]. Another study, again investigating Ag and AuNPs, distinguished AuNPs between 10 nm and 20 nm and 10 nm, 20 nm and 40 nm AgNPs with an *R*^2^ of 0.9982 for the relationship between size and mobility of the AgNPs. Here, a borate/Tris buffer system at pH 9 was employed to achieve CE separation in less than 10 min [46]. Both studies discussed here demonstrate proof of concept of the applicability of CE-ICP-MS to assess complex NP mixtures at environmentally relevant trace levels [36,46].

## 4. CE for NM Corona Characterisation

While the size, shape, charge, zeta potential and composition provide many critical physiochemical properties of a NM, in biological and environmental systems the dynamically formed surface coating, or the acquired biomolecule corona, also plays a vital role in how NMs are “seen” by cells [17,18,19,89,90] and organisms [91,92]. When a NM is exposed to a biological (or environmental) matrix such as blood, biomolecules present form a corona by binding to the surface of the NM, thereby modifying its exterior. It is generally considered that there are two types of corona: a hard corona which is long term and formed of proteins with a high binding affinity for the NM surface and, a soft corona which is more dynamic, with frequent exchange of molecules between surrounding/media and NM, formed of biomolecules with a lower affinity for the NM [18,20,21], or via protein–protein interactions with proteins in the hard corona. The properties of the corona are known to influence cellular uptake and clearance in addition to NM transport and biodistribution. A number of factors such as the surface chemistry and NM size are known to influence which proteins form the corona and their relative abundance [18,20,93]. Interestingly, the corona composition does not reflect the concentration of proteins found in the surrounding environment, rather, NMs will preferentially bind some proteins over others and even retain a “memory” of their previous environments [17,21]. This opens up the possibility to manipulate coronas to target specific cellular receptors in vivo in order to increase drug efficacy and reduce off-target toxicity and effects [17,18,19,20,90,94]. Conversely, the corona constituents accumulated from the NMs environment can also have negative implications, such as facilitating unintentional crossing of the blood–foetus or blood–brain barriers by NMs, or by eliciting their uptake into a range of environmental organisms and causing subsequent toxicity [91,95,96,97]. Further investigation into NM corona composition and dynamics will enable a much greater understanding of how this biological layer can manipulate NM distribution, toxicity and bioavailability. Specifically, focus on other constituents in addition to proteins, such as small molecules and carbohydrates, will provide new molecular insights and perhaps facilitate identification of signatures of uptake, and impact.

### 4.1. Protein Corona Characterisation

To date, the protein corona has been investigated using several methods. These include DLS to determine changes in NM size and zeta potential following exposure of biological media, UV-vis which characterises the changes in surface plasmon resonance or ITC to characterise the stoichiometry of individual protein–NM interactions [19,94] and CE-UV to investigate binding efficiency [98]. These techniques however are limited in that they can only detect changes and cannot identify what has been bound to the NM surface. Thus, while these techniques provide a very quick and simple check for the actual formation of the corona very little can be ascertained in terms of protein (biomolecule) composition and identity. To date, CE-UV has been used for the analysis of apolipoproteins in the corona of poly(methoxypolyethyleneglycol cyanoacrylate-*co*-hexadecyl cyanoacrylate) (PEG-PHDCA) NPs to investigate their ability to cross the blood–brain barrier. However, due to the limited sensitivity of the UV detector, low abundance apolipoproteins were poorly detected and the study itself was limited to known, well characterised proteins [99].

To elucidate more detailed and quantitative information on the protein corona, mass spectrometry techniques are required. Protein characterisation can be achieved in two ways: shotgun/bottom up or top down. Both of these methods have been used extensively in the field of proteomics and have been well described in the literature [100,101,102]. In brief, top-down analysis begins with an intact protein which can be fragmented once within the mass spectrometer and, unlike bottom up, can distinguish between some isoforms and post translational modifications [100,102]. However, to date, no NM corona had been characterised in this manner. A bottom up approach utilizes several wash steps with a phosphate, EDTA and sodium chloride buffer to remove the surrounding media and loosely associated molecules followed by a protease digest, typically trypsin, to produce a complex array of peptides which can be infused into the mass spectrometer directly or chromatographically separated to enhance peptide/protein coverage [20,21,103]. This method has been used successfully in several studies where the protein corona has either been digested on the NM, or extracted from the NM using SDS with the proteins intact and run on an SDS-Polyacrylamide gel electrophoresis (SDS-PAGE) gel before digestion and LC-ESI-MS/nLC-nESI-MS/MALDI-MS analysis [17,19,20,21,94]. To date, no comparison of these two sample preparation methods has been performed and as a result it is not known if the SDS extraction or on-particle digest fully removes the corona or if it leaves the hard corona intact or partially intact. Another area of interest for sample preparation is whether the hard and soft coronas can be analysed independently. One potential way to achieve this for a bottom up approach would be to vary the incubation time for the protein digest, for example a shorted incubation may allow the outermost layers of the corona to be digested and analysed before the more closely bound layer. Another possibility that would apply for both bottom up and top down approaches would be to use gradually increasing concentrations of SDS to elute more and more strongly bound proteins from the corona which could then be digested off particle or injected in this from, depending upon the desired proteomics approach. To comprehensively characterise the protein corona the quality of the sample preparation must be investigated. Further to identifying and quantifying the proteins that form the corona it would also be of significant interest to begin to investigate the conformation/interacting surface of the proteins that form the corona. One potential method to do this is hydrogen-deuterium exchange whereby the NM and its associated protein corona could be incubated in a D_2_O rich buffer allowing the buffer facing hydrogens to exchange for deuterium and the resulting protein analysed [100,104,105,106]. This would potentially improve the understanding of what aptamers are presented on the surface of the corona, which may elucidate the mechanism behind the formation of the corona and subsequent NM transport.

While conventional untargeted high resolution mass spectrometry (HRMS) based protein characterisation has typically been achieved using LC-ESI-HRMS/nLC-nESI-HRMS [107], the rise in popularity of capillary electrophoresis-high resolution mass spectrometry (CE-HRMS) offers a complimentary, near orthogonal, technique for the unbiased characterisation of the proteins in a NM’s corona [108,109]. Furthermore, the unique separation offered by CE enhances the detection and separation of small polar peptides, glycosylated and phosphorylated proteins from nL sample sizes, whilst also providing very low flow rates (<10 nL/min). This enables highly sensitive nESI-MS, when a sheath-less CE system is utilised [110,111,112,113]. To date, CE-MS has been successfully used in a wide range of proteomic studies investigating a variety of diseases such as cancer [114], kidney disease [115] and for protein characterisation in antibody-drug interactions [111,112,116] and this upward trajectory in CE-MS based proteomics is likely to continue. As such, CE-MS, with its unique separation and sensitivity, makes for an exciting new tool for the characterisation of the NM protein corona.

### 4.2. Small Molecule Corona Characterisation

**Figure 4:**
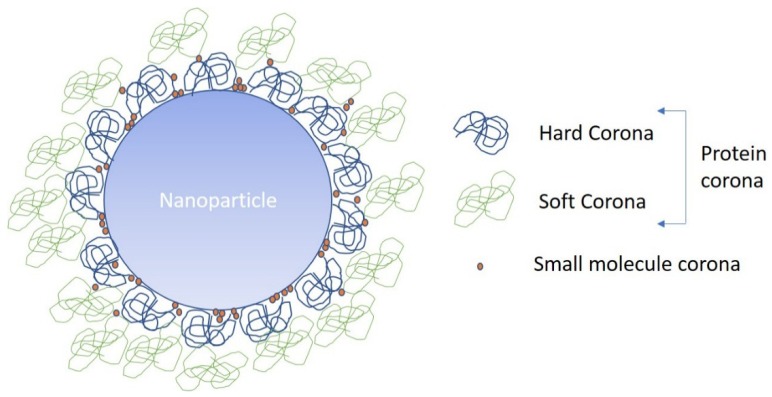
Schematic representation of the NP biomolecule corona, indicating the small molecules, which can squeeze in between the larger proteins forming the hard or tightly bound corona, and the loosely bound soft corona, typically held via protein–protein interactions and rapidly exchanging with the surrounding biomolecules.

While the protein corona has received a lot of interest, there has been little to no serious investigation into the ability of small molecules (<1000 Da) to bind to and affect the distribution and biological or environmental processing of NMs. It is already known that small molecules bind to NMs as this principle is the basis of increased sensitivity in SERS [117,118], and small molecule interactions with NMs have been proposed as a means to develop quantitative structure–activity relationships for NMs based on the concept of the Biological Surface Adsorption Index [119]. NMs have also been used to clean up small molecule contaminants, either through binding and subsequent removal of NMs, or via catalytic reactions to break down contaminants [120,121,122]. These studies show that NMs and small molecules can interact, suggesting that not only proteins may form the corona (Figure 4), but also other small molecules present in biological and environmental matrices. However, there have been no studies to determine which small molecules will bind, their associated NM binding dynamics or how these can influence NM behaviours such as cellular uptake, localisation, toxicity or formation of the protein corona. As such there is no currently used method for the extraction of the small molecule corona from the NM itself. Unlike in the determination of the protein corona, no digestion is required and as such following several washes with water small molecules may be extracted from the NM using an organic solvent such as methanol. However, due to the novel nature of this analysis, a study to develop a robust sample preparation method would be required to ensure that the full small molecule corona is being analysed.

As is necessary when characterising the protein corona, an untargeted, non-biased analysis of the small molecule corona is required. This approach, when used to study biofluids and tissues, has been termed metabolomics [123]. Traditionally, nuclear magnetic resonance (NMR), gas chromatography–mass spectrometry (GC-MS) and LC-MS have been used for metabolomic analyses [124,125,126]. Due to the small sample size, a miniaturised LC-MS approach may offer advantages, and nanoflow LC-nanosprayESI-MS has been used in metabolomics to improve sensitivity while using minimal sample volumes [127,128]. However, the much lower flow rates, and small sample volume required make CE-MS an interesting prospect for analyses of the small molecule NM corona, as sample volume is likely to be very limited. CE has been used over a number of years for metabolomic analysis with success, however it has yet to become a mainstream technique, due to limited experience in CE of many researchers and perceived poor reproducibility [129,130,131]. The use of modern CE instruments eliminates many of the concerns raised previously with regard to migration time reproducibility, with these systems now being on a par with LC approaches [132,133]. Typically, CE-MS is more widely used for the analysis of cations using normal polarity separation across the capillary, i.e., with the anode set to the end of the detector. Using a high EOF it is possible to also analyse anions, however typically the ionisation mode on the MS would need to be swapped to negative ESI mode. Even in this case, some anions would be lost as they migrate to the cathode faster than the rate of EOF [131]. However, a number of studies have developed methods to reverse the EOF in order to carry anions to the mass spectrometer inlet, enabling their detection with minimal loss [131,134]. The possibility of modern CE coupled to high resolution mass spectrometers, such as time of flight or orbitrap, offers the potential for a highly sensitive and reproducible technique to explore the as yet poorly studied small molecule NM corona.

## 5. Conclusions

The current studies involving the use of CE for the separation of NMs and NM mixtures indicate that there are significant advantages offered with this technique when compared to several more common characterisation tools, not least the wider range of physico-chemical parameters that can be determined/calculated simultaneously from a single measurement. CE separations can be applied to a wide range of NM compositions, sizes and shapes, and can be used to determine the absolute size, relative size, size distribution, composition, surface charge and zeta potential, the measurements of which have been demonstrated to compare favourably with current gold standard methods for determining each parameter individually. Due to this diverse range of capabilities, it is possible to use a single CE experiment to determine these properties in typically less than 10 min of experimental time, which provides significant advantages when compared to the wide range of instruments and expertise required for other methods such as DLS, FFF, TEM and UV-vis. Furthermore, CE-MS offers an exciting prospect for the characterisation of the NM corona, both proteins and small molecules. CE’s low volume sample requirement coupled to its unique separation ability and high sensitivity offers a complementary method for the analysis of the protein corona. These properties also make CE-MS an exciting prospect for exploring the role small molecules play in the NM corona, an area yet to be investigated.

## Figures and Tables

**Table 1:** Comparison of common tools to characterize NMs and their relative drawbacks compared to CE.

Technique	Property Measured	Advantages of Determining via CE	Refs
**Dynamic Light Scattering (DLS)**	Hydrodynamic diameter of a particle Zeta potential Electrophoretic mobility	Less expensive More versatile Able to analyse polydisperse samples or complex samples Accurate for the sizing of non-spherical particles	[4,5]
**Field Flow Fractionation (FFF)**	Separation technique that can separate materials over a wide colloid range	Less expensive Typically less than 20 min run times Very little loss of sample to capillary whereas significant losses to the FFF membrane can occur if improper sample preparation and method development is performed	[6,7,8]
**Transmission Electron Microscopy (TEM)**	Microscopy technique allowing for size and shape determination of electron dense materials	Less expensive Non-destructive Rapid (typically <20 min per sample) Minimal sample preparation required Relatively larger sample volume/amount analysed making analysis more representative Clear cut results with no user interpretation required thus reducing bias	[9,10]
**Ultra-Violet Visible Light Spectroscopy (UV/VIS)**	Spectroscopy technique able to quantitatively determine different analytes and biological macromolecules	Can be used as a separation technique	[5]

**Table 2:** NM characteristics that drive CE based separations and properties that can be determined using CE with optical or mass spectrometer detectors.

NP Characteristic Driving Separation	NP Properties that Can Be Calculated Using CE-UV/LIF/LLS	NP Properties that Can Be Calculated Uniquely by CE-_(SP)_ ICP-MS
Size	Relative size (using calibration curve)	Elemental composition
Shape	Zeta potential	Size
Cross sectional area	Surface charge density	Size distribution
Surface charge/functionalisation	Concentration	Trace level concentrations
Capping material		Particle number concentration (spICPMS)
Composition		

**Table 3:** Summary of CE methods used to date for NM characterisation.

NM Composition	NP Diameters (nm)	Capillary Material/Dimensions	Injection Pressure and Duration	Separation Voltage and Temperature	Separation Time (min)	Background Electrolyte	pH	Detection	Result	Ref
Gold	Not defined	BFS 50 µm × 30 cm	100 mBar 3 s	Not provided 25 °C	35	30 mM sodium phosphate in 20% EtOH	10.0	LIF 485/550 nm UV-Vis 214 nm	Baseline separation of a range of NPs	[41]
Gold	5.3, 12.1, 40.1, 59.9	BFS 75 µm i.d. × 33.5 cm	50 mBar 5 s	20 kV 25 °C	4	70 mM SDS, 10 mM CAPS	10.0	UV-Vis 520 nm	Size vs. Mt *R*^2^ 0.985. Good correlation between TEM and CE for size measurements	[38]
Gold	3.5, 6.5, 10.5	BFS 50 µm i.d. ×100 cm	4.9 kPa 100 s	10 kV 20 °C	25	20 mM NH_4_AC, 20 mM TRIS, 10 mM CAPS	8.5	ELSD	Non-baseline separation of NPs. Ability to distinguish between 3 NP sizes covering just a 7 nm size difference. Good correlation between CE and TEM	[42]
Gold	5, 10, 20, 40	Polyamide coated BFS µm i.d. × 36.5 cm to UV and 45 cm to C^4^D	50 mBar 12 s	20 kV	15	20 mM PIPES	7.4	UV-Vis 210/220/235 nm ICP-MS C^4^D	CE-ICP-MS LOD of 2 × 10^−15^ M. Conductivity not suitable as a detector for AuNPs	[14]
Gold	5.3, 40.1	BFS 75 µm i.d. × 25 cm	50 mBar 2 s	20 kV 25 °C	3	70 mM SDS, 10 mM CAPS	10.0	UV-Vis 520 nm	REPSM increases sensitivity. Addition of NaH_2_PO_4_ for reduced migration time	[32]
Gold	5, 20	BFS 75 µm i.d. × 55 cm	5 s	28 kV	6	50 mM TRIS	9.2	UV-Vis 520 nm	Separation of the two NP sizes. Ability to separate AuNPs from polystyrene NPs	[45]
Gold	5.2, 5.9, 7.2, 8.6, 14.6	BFS 75 µm i.d. × 27 cm	50 mBar 5 s	20 kV 25 °C	10	6 mM NH_4_Ac/acetic acid	5	UV-Vis 520 nm	Good correlation between size and mobility *R*^2^ 0.9745	[39]
Gold	5.3, 9.8, 19.0, 29.3, 41.2	BFS 75 µm i.d. × 43.1 cm	10 s	20 kV 20 °C	Not defined	70 mM SDS, 10 mM CAPS	10.0	UV-vis 546 nm	Good correlation between size and mobility *R*^2^ 0.99. Mobility RSD below 1% for 5.3 and 19 nm AuNPs	[31]
Gold	5, 10, 21.5, 30.2, 41.2	BFS 75 µm i.d.	50 mBar 3 s	18 kV 15 °C	<5	70 mM SDS, 10 mM CAPS	11.0	UV-vis	Strong linear relationship between NP size and mobility *R*^2^ 0.992. Electrophoretic mobility RSD <0.8%. Good correlation between CE and SEM methods for NP size determination	[60]
Gold	5, 40, 60	BFS 75 µm i.d. × 33.5 cm	50 mBar 50s	20 kV 25 °C	4	70 mM SDS, 10 mM CAPS	10	UV-vis 520 nm	Baseline separation of NPs with a *R*^2^ 0.99 for linearity of mobility and NP size. REPSM method utilized to improve sensitivity	[61]
Gold	10, 30, 60	Polyimide coasted fused silica capillary	50 mBar 5 s	30 kV	<11	70 mM SDS 10 mM CAPS	10	spICP-MS	Determination of Mt, size, PNC in a single analysis. Non-baseline CE separation due to broad particle size distribution. Strong linear relationship between particles injected and particles detected *R*^2^ ≥ 0.998	[52]
Gold Gold/Silver	17.2 60.1	BFS 75 µm i.d. × 25 cm	50 mBar 50 s	20 kV 25 °C	5	40 mM SDS, 10 mM CAPS	10.0	UV-vis 520 nm	Baseline separation between the AuNPs. REPSM method utilized to improve sensitivity	[30]
Gold and silver	Au: 5, 10, 20, 50 Ag: 7, 30	Polyamide coated fused silica capillary 75 µm i.d. × 70 cm	50 mbar 3 s 20 kV 8 s	29 kV	<10	60 mM SDS, 10 mM CAPS	10	ICP-MS	Distinguished between AuNPs and AgNPs. Strong correlation between mass spectrometer response and NP size *R*^2^ = 0.999	[36]
Silver	17, 49.7	BFS 75 µm i.d. × 40 cm	50 mBar 1 s	20 kV 15 °C	<20	20 mM SDS, 10 mM TRIS	8.5	UV-Vis 350, 395 440 nm	Baseline separation of the 2 NPs. Non-baseline separation of NP (sphere) and NM (rod)	[63]
Silver and gold	10, 20, 40 10 20	BFS 75 µm i.d. × 60 cm	50 mBar 15 s	25 kV 25 °C	10	10 mM Tris, 10 mM H_3_BO_3_, 10 mM Na_2_B_4_O_7_	9.0	ICP-MS	Non-baseline separation of the 3 NPs however, good linear relationship between size and mobility *R*^2^ 0.9982 Size determination compared favourably to DLS and TEM	[46]
Silver	20, 40, 60	Polyamide coated fused silica capillary, 75 µm i.d. × 70 cm	50 mBar 3 s REPSM up to 150 s	20 kV	<30	60 mM SDS 10 mM TRIS	10	spICP-MS	REPSM method used to improve sensitivity. Good correlation between NPs detected and injection time *R*^2^ > 0.99. Good linearity for mobility and separation voltage *R*^2^ > 0.99. LOD < 1 µg/L	[53]
Silver	Citrate capped: 20, 40 60 PVP capped: 40, 60 PEG coated: 40 BPEI coated: 40	Polyimide coated fused silica 75 µm i.d. × 70 cm	50 mBar 10 s then −20 kV REPSM	20 kV	<30	60 mM SDS 10 mM CAPS	10	spICP-MS	Separation of NP coating by CE prior to spICP-MS detection. REPSM method used to improve sensitivity	[54]
Fullerenes	C3 and DF1	BFS MEKC BFS dynamic coating for CZE 50 µm × 30 or 50 cm	0.5 PSI 20 s	+22 kV BFS −22 kV dynamic coating	15	MEKC: 150 mM SDS, 10 mM Sodium tetraborate CZE: 10 Mm Sodium tetraborate	9.2	DAD 250 nm	LODs of between 0.6 and 6 µg/mL	[64]
Carbon	nd	BFS 50 µm × 50 cm	0.5 PSI 5 s	25 kV 22 °C	60	80 mM glycine	9.9	DAD 230 nm	Separation of different carbon NMs achieved	[65]
PVP stabilized SWNT	nd	BFS 75 µm × 37.5 cm	500 mBar 2 s	15 kV	<35	50 mM Trizma base 0.5% SDS	nd	Raman	Separated SWNT based upon length, diameter and cross-sectional area	[66]
SWNT	Length 0.2–1.2 0.5–2.5 2–4 1.8–10	BFS 75 µm × 75 cm (UV) 25 cm Ramen	100 mBar 30 s	5 kV	20	50 mM Trizma base 0.5% SDS		UV 360 nm Ramen	Separated SWNT based upon length. Improved size selectivity than FFF and size exclusion chromatography	[67]
Graphene oxide (GO) and reduced graphene oxide (CCG)	nd	Polyimide coated BFS 75 µm × 41.5 cm	50 mBar 5 s	15 kV 22 °C	15	250 µM tetrapropylammonium hydroxide	10.4	UV GO 230 nm CCG 270nm	Ability to differentiate GO and CCG demonstrated	[68]
SWNT MWNT	SWNT 1.2–1.5 nm and 0.7–1.2 nm diameter 2–5 and 2–20 µm MWNT 20–50 nm diameter, −20 µm	BFS 75 µm × 47 cm	0.5 PSI 10 s	15 kV	10	5 mM NH_4_AC with 0.025% HPMC	8.03	DAD 240 nm	Distinguished SWNTs and MWNTs based upon size and volume. Mt reproducibility RSD 2.7–5.4%. Peak area reproducibility RSD 3.7–7.8%	[69]
Fullerenes	C60 C70	BFS 75 µm × 28 cm	20 mBar and gravity fed	10 kV 20 °C	26	10 mM borate phosphate with 100 mM SDS	9.5	UV 254 nm	Separation of C_60_ and C_70_	[70]
Graphene oxide (GO)	nd	BFS 75 µm × 50 cm	200 mbar 40 s	10 kV	45	50 mM borate	11	UV 280 nm	GO sheets separated based upon size and stacking	[71]
Silica	20, 50, 100	BFS 50 µm × 50 cm	50 mBar 10 s	27 kV 20 °C	<20	3 mM NH_4_AC 1% MeOH	6.9	ELSD	Strong linear relationship between peak area and NP concentration *R*^2^ 0.999 LOD 1.08 ng/nL Peak area RSD <6%. Near baseline separation of the 3 NP sizes	[34]
Silica	7, 12, 22	BFS 75 µm × 29.2 cm	0.1 PSI 0.2 6 s	7 kV 15, 20, 25 °C	40	20, 30, 40, 50, 60 mM Borate	nd	UV/TDA	Zeta potential, surface charge density and hydrodynamic sized determined	[47]
Polystyrene	20, 50, 155, 300	BFS 75 µm i.d. × 55 cm	5 s	28 kV	6	50 mM TRIS	9.2	UV 520 and 254 nm	Baseline separation of the 4 NP sizes. Ability to separate polystyrene NPs from AuNPs	[45]
Polystyrene	55 and 70	BFS 75 µm i.d. × 66.5 cm	17 mBar 6 s	7 kV 25 °C	35	12.7 mM Borate	9.2	UV-Vis/TDA	CE-TDA correlated with TDA and DLS readings	[51]
Polystyrene	39, 72, 132, 308, 488, 683	0.5 mM CTAB treated BFS 50 µm i.d. × 47.6 cm	30 kV 1 s	30 kV	5	1 mM ACES	5.8	UV-Vis 225 nm	Separation of the 6 NP sizes. Linear relationship between NP size and mobility *R*^2^ 0.903 calculated manually from data presented. Mt RSD of 1.4%	[33]
Polystyrene	100, 180 800	BFS 30 µm i.d. × 10 cm	HPLC injector used	10 kV and pressure 1.1–3 kgf/cm^2^	2	10 mM Borate	8.2	UV-Vis 210 nm	Electrophoretic mobility was augmented by applying pressure to capillary	[35]
Polystyrene	50, 102, 204, 404, 600	BFS 75 µm i.d. × 50 cm	1.38 kPa 10 s	−30 kV 30 °C	<15	5 nM phosphate buffer	9	UV-Vis 200 nm	Separation of the 50, 102, 204 and 404 nm NPs	[72]
Iron	HNO_3_ stabilized: 6.8, 8.9, 10.6 Citrate stabilized: 7.0, 8.9, 10.3 TMAOH stabilized: 6.4, 7.9	50 µm i.d. × 26.5 cm HPC coated BFS PB coated BFS DDABr coated BFS BFS	30 mBar 3 s	BFS and HPC coated 10 kV PB and DDABr coated −10 kV 25 °C for all	<15 PB <5 PB and DDABr BFS not defined	HPC coated BFS for HNO_3_ stabilized FeNP 10.5 mM alanine and 10 mM HCl PB and DDABr coated 10 mM HCl Citrate stabilized FeNP on BFS: 5.7 mM TMAOH and 2.4 mM citrate TMAOH stabilized FeNP: 5 mM TMAOH	2.9 2.0 6.1 nd	UV-Vis 200 and 254 nm	Characterized mobility of FeNP in BFS capillary with different coatings. Size based separation for <11 nm FeNPs	[73]
Iron	All the same undefined size with different surface charge densities	50 µm i.d. × 26.5 cm DDABr coated BFS	20 mBar 2 s	−10 kV 25 °C	Not defined	106.6 mM Tris 100 mM HCl	8	UV-Vis 200 and 254 nm	Separation driven by surface charge density. Surface charge density determined in a more reproducible manner than the ninhydrine colorimetric assay	[74]

ACES: *N*-2-aminoethanesulphonic acid; BFS: bare fused silica; CAPS: *N*-cyclohexyl-3-aminopropanesulphonic acid; CE-TDA: capillary electrophoresis-Taylor dispersion analysis; CE-ICPMS: Capillary electrophoresis-inductively couple plasma mass spectrometry; DDABr: didodecyldimethylammonium bromide; EtOH: Ethanol; Fe NP: Iron nanoparticle; HPC: hydroxypropyl cellulose; i.d.: internal diameter; LOD: limit of detection; MeOH: methanol; MWNT: multi-walled nanotubes; NH_4_AC: ammonium acetate; NM: nanomaterial; NP: nanoparticle; PB: hexadimethrine bromide/polybrene; PIPES: piperazine-*N*,*N*′-bis(2-ethanesulphonic acid); Tris: *tris*(hydroxymethyl)aminomethane; REPSM: reversed electrode polarity stacking mode; RSD: relative standard deviation; SDS: sodium dodecyl sulphate; SWNTs: single-walled nanotubes; TMAOH: tetramethylammonium hydroxide.

## Abbreviations

APTES	3-(aminopropyl)triethoxysilane
BFS	bare fused silica
BGE	background electrolyte
BPEI	branched polyethyleimine
CAPS	3-cyclohexylamino-1-propanesulphonic acid
CCG	chemically converted graphene
CE	capillary electrophoresis
CE-MS	capillary Electrophoresis–Mass Spectrometry
CGE	capillary gel electrophoresis
cIEF	capillary isoelectric focussing
CNT	carbon nanotubes
CTAB	cetrimonium bromide
CZE	capillary zone electrophoresis
DDABr	didodecyldimethylammonium bromide
DLS	dynamic light scattering
EDTA	ethylenediaminetetraacetic acid
EOF	electroosmotic flow
FFF	field flow fractionation
GO	graphene oxide
HCl	hydrochloric acid
HPC	hydroxypropyl cellulose
HRMS	high resolution mass spectrometry
ICP-MS	inductively coupled plasma–mass spectrometers
ITC	isothermal titration calorimetry
ITP	isotachophoresis
LC-MS	liquid chromatography–mass spectrometry
LC-ESI-MS	liquid chromatography–electrospray ionisation–mass spectrometry
LIF	laser induced fluorescence
LLS	laser light scattering
LOD	limit of detection
LOQ	limit of quantification
MALDI-MS	matrix assisted laser desorption ionisation–mass spectrometry
MEKC	micellar electrokinetic chromatography
MWNT	multi-wall nanotubes
nESI	nanoelectrospray ionisation
nLC-nESI-MS	nanoliquid chromatography–nanoelectrospray ionisation–mass spectrometry
NM	nanomaterials
NMR	nuclear magnetic resonance
NP	nanoparticle
PB	polybrene
PEG	polyethylene glycol
PVP	polyvinylpyrrolidone
PEOS	2-[methoxy(polyethyleneoxy)propyl]trimethoxysilane
PEG-PHDCA	poly(methoxypolyethyleneglycol cyanoacrylate-*co*-hexadecylcyanoacrylate)
PNC	particle number concentration
REPSM	reversed electrode polarity stacking mode
RSD	Relative Standard Deviation
SDS	sodium dodecyl sulphate
SDS-PAGE	sodium dodecyl sulphate-Polyacrylamide gel electrophoresis
SERS	surface enhanced Raman spectroscopy
spICP-MS	single particle inductively coupled plasma–mass spectrometers
SPIONs	superparamagnetic iron nanoparticles
SWNT	single-wall nanotubes
TDA	Taylor Dispersion Analysis
TEM	transmission electron microscope
TMAOH	tetramethylammonium hydroxide
Tris	tris(hydroxymethyl)aminomethane
UV	ultraviolet
